# A rare huge bladder inflammatory myofibroblastic tumor treated by en bloc resection with diode laser: a case report and literature review

**DOI:** 10.3389/fonc.2024.1327899

**Published:** 2024-03-11

**Authors:** Huisheng Yuan, Zilong Wang, Jiaxing Sun, Junhao Chu, Shishuai Duan, Muwen Wang

**Affiliations:** ^1^ Department of Urology, Shandong Provincial Hospital Affiliated to Shandong First Medical University, Jinan, China; ^2^ Department of Andrology, The Seventh Affiliated Hospital, Sun Yat-sen University, Shenzhen, China; ^3^ Department of Urology, Shandong Provincial Hospital, Cheeloo College of Medicine, Shandong University, Jinan, China

**Keywords:** inflammatory myofibroblastic tumor, en bloc resection, 1470 nm diode laser, second transurethral resection, bladder cancer, case report

## Abstract

**Background:**

Inflammatory myofibroblastic tumor (IMT) is a rare neoplasm with malignant potential. Bladder IMT is even rarer and mainly treated by surgical resection However, partial or radical cystectomy would affect the quality of life of patients due to major surgical trauma, and classical TURBT is hard to avoid intraoperative complications including obturator nerve reflex and bleeding etc. Therefore, the safe and effective better choice of surgical approaches become critical to bladder IMT.

**Case presentation:**

A 42-year-old male patient was admitted to the department of urology with persistent painless gross hematuria for more than 10 days without the presentation of hypertension. Preoperative routine urine examination of red blood cells was 7738.9/HPF (normal range ≤ 3/HPF). CTU indicated a space occupying lesion (6.0 cm×5.0 cm) in the left posterior wall of the bladder with heterogeneous enhancement in the excretory phase. MRI also indicated bladder tumor with slightly equal SI on T1WI and mixed high SI on T2WI (6.0 cm×5.1cm×3.5cm) in the left posterior wall of the bladder. En bloc resection of bladder IMT with 1470 nm diode laser in combination of removing the enucleated tumor by the morcellator system was performed. Postoperative pathological examination revealed bladder IMT, with IHC positive for Ki-67 (15-20%), CK AE1/AE3, SMA, and Desmin of bladder IMT and negative for ALK of bladder IMT as well as FISH negative for ALK gene rearrangement. Second TUR with 1470 nm diode laser was performed within 6 weeks to reduce postoperative risk of recurrence due to highly malignant potential for the high expression of Ki-67 (15-20%) and negative ALK in IHC staining. The second postoperative pathology report showed chronic inflammation concomitant with edema of the bladder mucosa without bladder IMT, furthermore no tumor was observed in muscularis propria layer of bladder. No recurrence occurred during the period of 24-month follow-up.

**Conclusion:**

En bloc resection of bladder IMT in combination of the following second transurethral resection with 1470 nm diode laser is a safe and effective surgical approach for the huge bladder IMT with highly malignant potential.

## 1 Introduction

Bladder inflammatory myofibroblastic tumors (IMTs), which originate from the mesenchymal tissue, are rare neoplasms with malignant potential (1). Since the bladder IMT was first described in 1980, only more than 100 cases have been reported worldwide (2). The exact etiology of bladder IMT remains uncertain, and its most clinical features are painless hematuria, urine frequency, urgency etc. (3).

Transurethral resection of bladder tumor (TURBT), partial cystectomy and radical cystectomy are the main treatment of choice for bladder IMT (4). However, partial or radical cystectomy may affect the quality of life of these patients due to major surgical trauma and serious complications. However, classical TURBT may arouse an obturator nerve reflex, even perforation and bleeding etc. Therefore, the safe and effective better choice of surgical approaches become critical to bladder IMTs.

We first described a case of a 42-year-old male patient with huge bladder IMT treated by en bloc resection of bladder tumor (EBRBT) with 1470 nm diode laser, in addition of second transurethral resection (TUR) with 1470 nm diode laser followed within 6 weeks. During 14-month follow-up, the patient showed no recurrence by MRI and cystoscopy. The objective of this case report was for the first time to evaluate the safety and efficacy of EBRBT and second TUR with 1470 nm diode laser for the treatment of huge bladder IMT.

## 2 Case description

### 2.1 Preoperative condition

**Figure 1:**
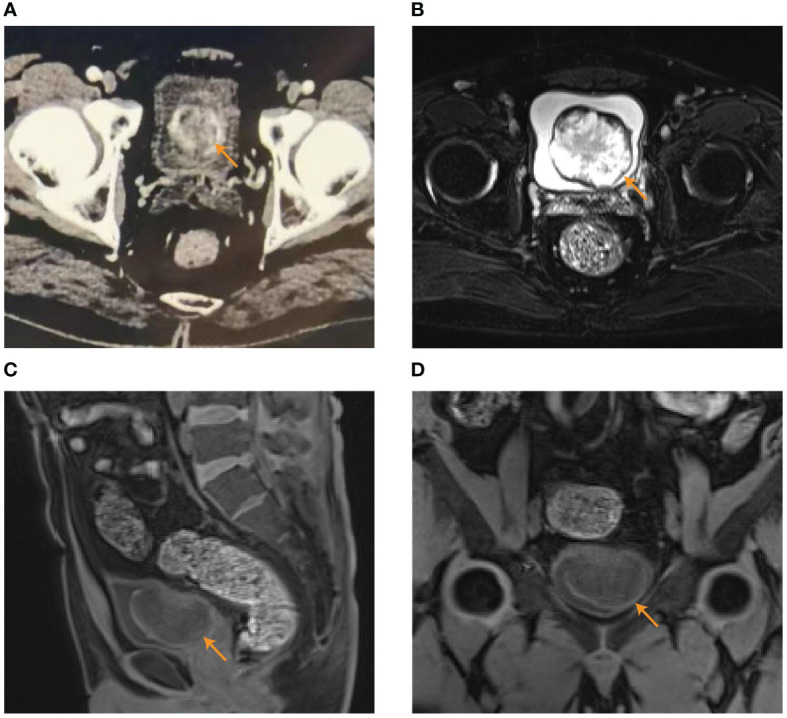
The results of preoperative examination. **(A)** CTU indicated a space occupying lesion (6.0 cm×5.0 cm) in the left posterior wall of the bladder with heterogeneous enhancement in the excretory phase. **(B–D)** MRI indicated bladder tumor (6.0 cm×5.1cm×3.5cm) with mixed high SI on T2WI in transverse plane **(B)** and slightly equal SI on T1WI in sagittal position **(C)** and coronal position **(D)**.

The 42-year-old male patient was admitted to the department of urology on 25th December 2021 with persistent painless gross hematuria for more than 10 days without the presentation of hypertension. The patient had the history of diabetes mellitus treated by Dimethylbiguanide for more than 2 years. Clinical laboratory tests including blood routine examination, serum electrolytes, liver function, blood lipid profile, and renal function were normal. Routine urine examination of red blood cells was 7738.9/HPF (normal range ≤ 3/HPF). The computed tomography urography (CTU) scan indicated a space occupying lesion (6.0 cm×5.0 cm) in the left posterior wall of the bladder with heterogeneous enhancement in the excretory phase (**Figure 1A**). Magnetic resonance imaging (MRI) also indicated bladder tumor with slightly equal signal integrity (SI) on T1-weighted imaging (T1WI) and mixed high SI on T2-weighted imaging (T2WI) (6.0 cm×5.1cm×3.5cm) in the left posterior wall of the bladder (**Figures 1B–D**). The preoperative diagnosis was huge bladder tumor.

### 2.2 En bloc resection of bladder tumor and postoperative pathological results

**Figure 2:**
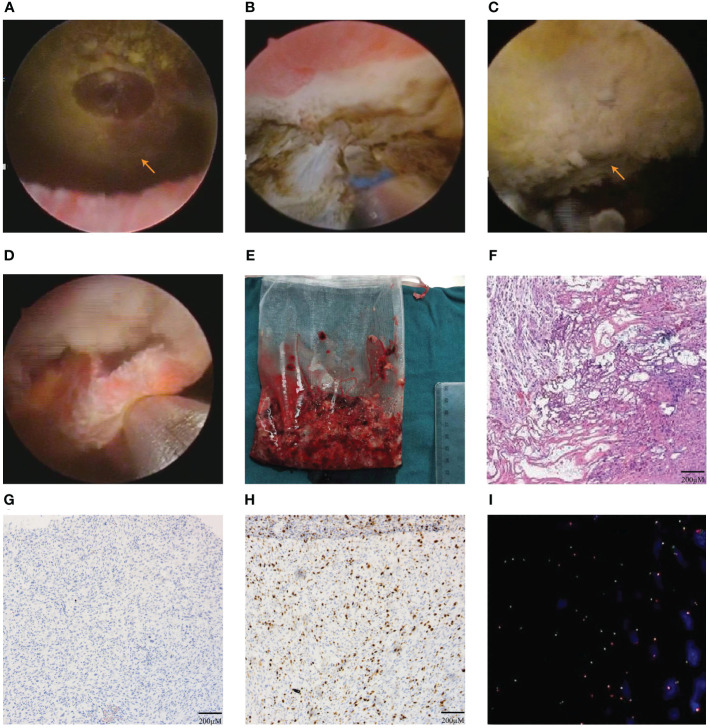
The En bloc resection of bladder tumor (EBRBT) and pathologic results. **(A)** Intraoperative cystocopic view of the huge bladder IMT. **(B)** At the base of the tumor, the circumferential margin was incised initially with 1470 nm diode laser to access the detrusor muscle layer. **(C)** The whole tumor was en bloc resected. **(D, E)** The enucleated en bloc tumor was removed by the morcellator system. **(F)** The result of first postoperative pathology with HE staining in bladder IMT. **(G–I)** The results in IHC staining of ALK(-) **(G)**, Ki-67+(15-20%) **(H)** and FISH of ALK gene rearrangement(-) **(I)**.

After adequate preoperative preparation, the patient received EBRBT on December 28^th^, 2021.The patient was placed in lithotomy position under general anesthesia. The 1470 nm diode laser system used 100 W for cutting and 30 W for coagulation. The diode laser energy with a wavelength of 1470 nm was delivered by a 600 µm fiber through a 26 F continuous-flow cystoscopy (Batch no.DQH-111, Hawk, Hangzhou, China). Firstly, we performed a thorough cystoscopic examination of the bladder and located an about 6.0×5.0cm polypoid tumor in the left posterior wall of bladder (**Figure 2A**). Then, the circumferential margin was marked about 2cm away from the gross margin of the tumor. The revealed sub-mucosal blood veins were then precoagulated. The circumferential margin was incised down to the detrusor muscle layer, causing the entire tumor to retract towards the center upon releasing the mucosa and submucosa. (**Figure 2B**). Subsequently, the tumor base can be accessed using both antegrade and retrograde approaches, employing a combination of sharp and blunt dissection techniques. The space between the layers of muscle was dissected sharply by laser. The tumor base was pushed and cut by laser till the submucosa and the connective tissue layers were exposed by the tip of the laser resectoscope sheath. The muscle fiber was separated by laser from the tumor’s base at the visible anatomical level until the whole tumor was en bloc resected (**Figure 2C**). The enucleated tumor was removed by the morcellator system (**Figures 2D, E**). The wound surface was examined carefully to ensure no bleeding. During the operation procedure, there were no significant complications, such as massive bleeding, bladder perforation and obturator nerve reflex. The patient received continuous bladder irrigation for 2 days and pirarubicin intravesical infusion therapy on postoperative day 1 and 7. Postoperative pathological examination revealed bladder IMT (**Figure 2F**), with immunohistochemistry (IHC) positive for Ki-67 (15-20%), cytokeratin AE1/AE3 (CK AE1/AE3), smooth muscle actin (SMA), and Desmin of bladder IMT and negative for anaplastic lymphoma kinase (ALK) of bladder IMT as well as fluorescence *in situ* hybridization (FISH) negative for ALK gene rearrangement (**Figures 2G–I**, **Supplementary Figures 1A–C**).

### 2.3 The second TUR and postoperative pathological results

**Figure 3:**
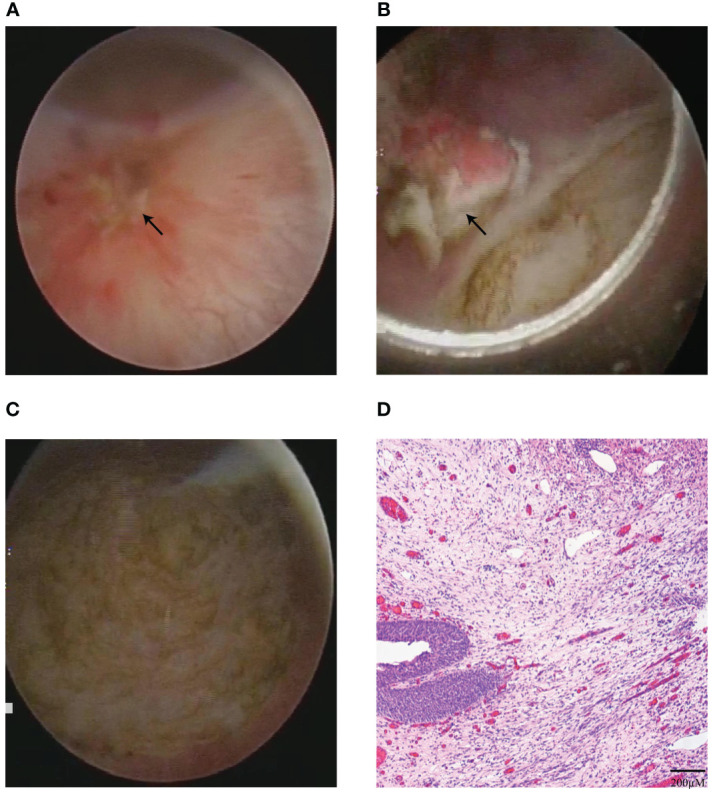
The second TUR and postoperative pathology results. **(A)** Intraoperative cystoscopic view of the surgical scar and inflammatory edema areas before second TUR. **(B, C)** The surgical scar and inflammatory edema areas were completely enucleated in second TUR with 1470 nm diode laser **(B)**, and surgical site after second TUR with 1470 nm diode laser **(C)**. **(D)** The result of second postoperative pathology with HE staining indicated chronic inflammation.

Due to the potential recurrent risk of bladder IMT for the high expression of Ki-67 (15-20%) and negative ALK in both IHC staining and FISH, second TUR was performed with 1470 nm diode laser within 6 weeks on February 8^th^, 2022. The procedure of second TUR was similar to the first en bloc resection of bladder IMT. Intraoperative cystoscopic examination showed that the surgical scar and inflammatory edema area were visible on the left posterior wall of the bladder (**Figure 3A**). Then, the circumferential margin was marked about 2cm from the scar and inflammatory edema area and incised initially to reach the detrusor muscle layer. The muscle fiber was cut until the entire scar and inflammatory edema area were enucleated (**Figures 3B, C**). The second postoperative pathology report showed chronic inflammation concomitant with edema of the bladder mucosa without bladder IMT, furthermore no tumor was observed in muscularis propria layer of bladder (**Figure 3D**).

### 2.4 The postoperative follow-up conditions

**Figure 4:**
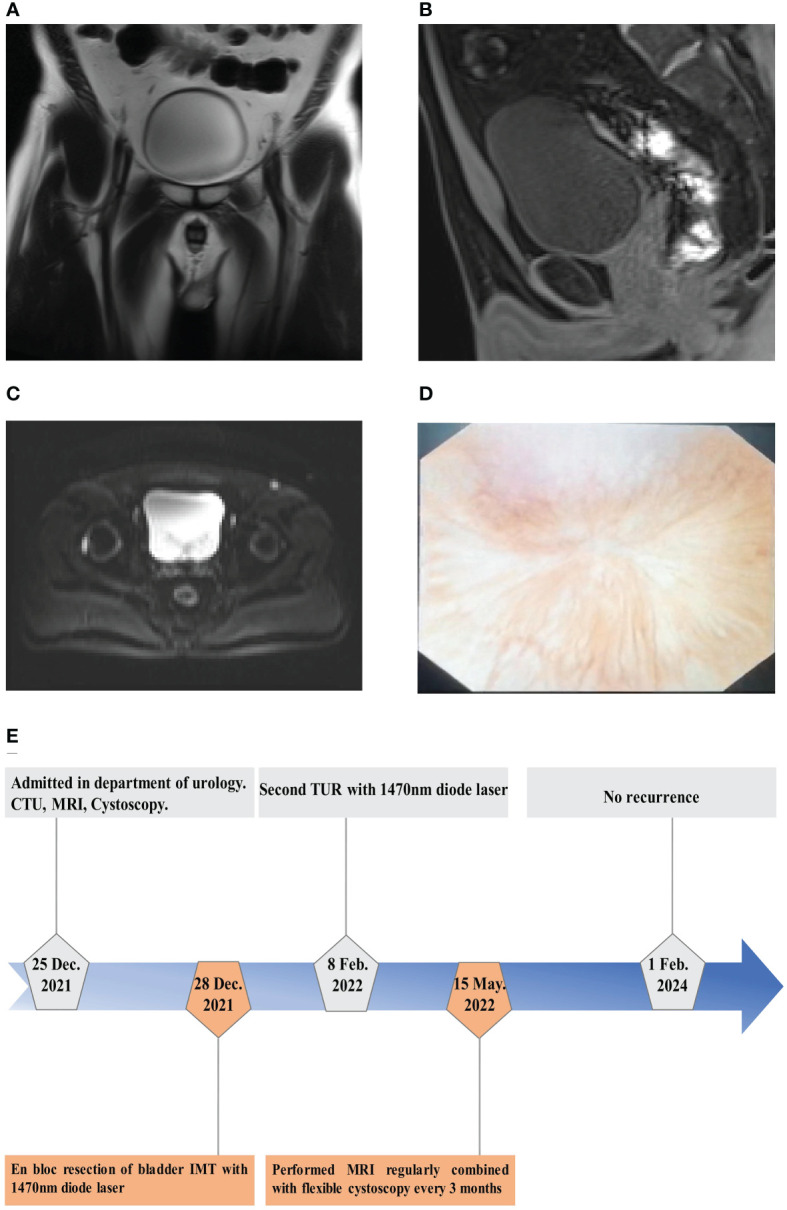
The results of follow-up examination after operation. **(A–C)** MRI showed no tumor recurrence on T2WI in coronal position **(A)** and transverse plane **(C)** and no tumor recurrence on T1WI in sagittal position **(B)** on postoperative month 14. **(D)** Flexible cystoscopy showed the performance of surgical wound healing on postoperative month 24. **(E)** The flowchart of timeline for diagnosis and treatment process.

During the 24-month period of follow-up after the following second TUR, the patient was performed MRI regularly combined with flexible cystoscopy every 3 months after the first EBRBT over 1 year. On postoperative month 14, the result of MRI showed no recurrence or distal metastasis of bladder IMT (**Figures 4A–D**). And flexible cystoscopy also showed no recurrence until February 1^st^, 2024. The flowchart of the timeline for the diagnosis and treatment schedule is shown in **Figure 4E**.

## 3 Discussion

IMTs are rare neoplasms of intermediate malignancy characterized by the predominant presence of a myofibroblastic mesenchymal spindle cell proliferation associated with inflammatory infiltration histologically and have a risk of local recurrence (5). IMTs mainly affect children and young adults and most frequently affected organs are lung and orbit (6), but IMT located in urogenital system is rare while bladder IMT is even rarer (1).

Considering the huge size (>5cm) of the bladder IMT (7), morcellator system was firstly introduced to remove the whole enucleated bladder IMT. Postoperative pathological findings showed bladder IMT with malignant potential, while immunohistochemistry indicated that the Ki-67 index was 15-20% and ALK FISH was negative. In order to avoid recurrence, the patient received second TUR with 1470 nm diode laser within 6 weeks and postoperative pathological showed no residual tumor while chronic inflammation of the bladder scar presented. The patient received MRI regularly and flexible cystoscopy every 3 months for more than 1 year and showed no recurrence lesions of bladder IMT during the period of 24-month follow-up.

Surgical resection with a negative margin is the mainstay of treatment for bladder IMT (8). Utilizing EBRBT specimens reduced diagnostic time and decreased inter-observer variability in T1 sub-staging compared to TURBT specimens (9, 10). With the development of laser technology, en bloc resection of bladder tumor (EBRBT) with 1470 nm diode laser offers a good alternative in the treatment of bladder tumor diseases. Compared with the traditional TURBT procedure, EBRBT with 1470 nm diode laser has the characteristics of less bleeding, clear operative field and easy to grasp the cutting depth, which can effectively reduce the occurrence of complications, length of hospitalization and recovery time (11). In this case, EBRBT with 1470 nm diode laser was applied for the first time to treat bladder IMT, and morcellator system was firstly used to remove the entire tumor, which reduce the risk of tumor dissemination. Thus, EBRBT with 1470 nm diode laser can serve as an efficient and feasible option for the treatment of the patient with huge bladder IMT.

IMT is considered as a member of the broad family of ‘Fibroblastic/Myofibroblastic Tumors ‘by National Comprehensive Cancer Network (NCCN) guidelines (version 2023). Pseudosarcomatous myofibroblastic proliferation (PMP) stands out as the most prevalent mimic of IMT. These entities exhibit analogous histological features, including the proliferation of elongated spindle cells, infiltrative growth, and a myxoid or edematous stroma, accompanied by a pronounced inflammatory component. Nevertheless, PMP is typically encountered shortly following instrumentation or in the context of local injury (12). Otherwise, differentiating these indolent lesions from malignant spindle cell tumors, such as leiomyosarcoma and sarcomatoid carcinoma, is of utmost importance. Histologically, IMTs are characterized by the presence of plump, stellate, or spindle cells arranged in a fascicular pattern (13). In comparison to bladder cancer, bladder IMT is frequently identified in the younger population (14). The imaging study reveals the presence of an infiltrating mass. However, it does not permit a conclusive differential diagnosis of the lesion, owing to its resemblances to malignant neoplasms. Thus, the confirmation of diagnosis relies on biopsy and subsequent histological analysis (1). During cystoscopy, bladder IMT manifested as isolated intraluminal polypoid masses (12), which facilitates its distinction from bladder cancer.

Bladder IMTs were defined as intermediate neoplasms by NCCN guidelines (version 2023). Although GASS et al. (15) reported that only 5 of 182 patients (2.7%) with IMTs developed local recurrences, there were still no clear epidemiological characteristics to be illustrated for lack of sufficient studies. Prognostic markers such as Ki-67 and ALK are needed for IMTs. Ki-67 is a reliable marker of cell proliferation, which can indicate the proliferation and malignancy potential of tumor cells. Culpan et al. (16) reported that patients whose Ki-67 index ≥15% had shorter progression-free, cancer-specific, and recurrence-free survival. Inamdar et al. (2) also reported that the Ki-67 labeling index in the resected specimens collected after IMT recurrence was higher than the Ki-67 labeling index in the specimen collected at the time of initial presentation. In our case, the bladder IMT with Ki-67 positive (15-20%) was considered a higher risk of recurrence.

Anaplastic lymphoma kinase (ALK) gene is located on chromosome 2p23, which is the main gene involved in the development of IMT and approximately 50% IMTs are in-volved in clonal rearrangements of ALK gene leading to the overexpression of ALK (17). Commonly, Fluorescence *in situ* hybridization (FISH) is the most popular method to detect rearrangement of ALK gene. ALK p80 or ALK D5F3 is ALK protein sensitive to IHC staining (18). However, for patients with either IHC negative or FISH negative for ALK, the malignant potential of bladder IMT cannot be excluded, while pathomorphology is more important in the diagnosis of IMT (4). For the atypical patients whose results of ICH are inconsistent with FISH, it would be best to perform Next-generation Sequencing (NGS) to make a more definite diagnosis (8). In the present case, although the IHC and FISH results of ALK in this patient were negative, while pathomorphology confirmed bladder IMT finally. Crizotinib is a Tyrosine Kinase Inhibitor (TKI) targeted ALK which not only has high anti-tumor activity against ALK-positive IMT, but also can reduce the tumor volume of ALK-negative patients (19), indicating that ALK-targeted drugs may be a treatment option for such kind of bladder IMT. Alectinib, originally designed for ALK fusion-positive non-small cell lung cancer (NSCLC), has been recently incorporated into the NCCN guidelines (version 2023) for ALK-positive inflammatory myofibroblastic tumors (20). It is worth noting that absent ALK expression of IMT has a relatively higher risk of distant metastasis (21). According to the study of Khondakar et al. (22) and Kim et al. (23), patients with bladder IMT of absent ALK expression both had tumor metastasis. In this case report, the patient has a relatively higher risk of distant metastasis because of IHC negative for ALK and FISH negative for rearrangements of ALK gene.

Secondary transurethral resection (TUR) is of great significance in preventing recurrence of bladder tumor. There was a 51% risk of tumor residue and 8% risk of underestimation of pathological stage after TURBT for T1 tumors (24). European Association of Urology (EAU) guideline recommends a second TUR within 6 weeks after the first TURBT especially for T1G3 non-muscle invasive bladder cancer (NMIBC) (25). Askin et al. also re-ported that second TUR in NMIBC patients had recurrence-free survival (RFS), progression-free survival (PFS), and the long-term overall survival (OS) advantages and second TUR was recommended as a routine treatment for all T1 stage NMIBC patients with life expectancy of at least 10 years (26). This case report showed that the patient performed second TUR with 1470 laser to prevent recurrence because of IHC positive for Ki-67 (15-20%) and negative for ALK with relatively high risk of recurrence and distant metastasis. During the follow-up period, the patient performed MRI regularly combined with flexible cystoscopy every 3 months for the first time. Therefore, the following second TUR by 1470 nm diode laser was the recommended approach to maintain the efficacy of EBRBT.

This study has the following limitations: Firstly, the time of follow-up is not long enough to evaluate the prognosis of bladder IMT although the en bloc resection of bladder tumor (EBRBT) with 1470 laser was feasible. Moreover, longer follow-up was required. Secondly, the result of IHC is ALK (-) and ALK gene rearrangement is negative by FISH, but the patient refused to performed NGS which was recommended to perform for these patients with IHC negative for ALK as a result of heavy economic burden. Thirdly, while uncontrolled hematuria was concerned if clinic biopsy, no biopsy was performed during the cystoscopy prior to surgery.

## 4 Conclusion

En bloc resection of bladder IMT as a rare tumor and following second TUR by 1470 nm diode laser is a safe and effective surgical procedure for bladder IMT with highly malignant potential.

## Data availability statement

The original contributions presented in the study are included in the article/**Supplementary Material**. Further inquiries can be directed to the corresponding author.

## Ethics statement

The studies involving humans were approved by Biomedical Research Ethic Committee of Shandong Provincial Hospital. The studies were conducted in accordance with the local legislation and institutional requirements. The human samples used in this study were acquired from a by- product of routine care or industry. Written informed consent for participation was not required from the participants or the participants’ legal guardians/next of kin in accordance with the national legislation and institutional requirements. Written informed consent was obtained from the individual(s) for the publication of any potentially identifiable images or data included in this article.

## Author contributions

HY: Writing – original draft, Writing – review & editing, Formal analysis, Funding acquisition. ZW: Conceptualization, Supervision, Writing – original draft, Writing – review & editing. JS: Investigation, Software, Validation, Writing – review & editing. JC: Data curation, Formal analysis, Writing – review & editing. SD: Data curation, Formal analysis, Project administration, Writing – review & editing. MW: Funding acquisition, Supervision, Validation, Writing – original draft, Writing – review & editing.

## Supplementary material

Supplementary Figure 1 The first postoperative pathology results. **(A–C)** The results in IHC staining of CK(AE1/AE3) (+) **(A)**, Desmin(+) **(B)**, and SMA-auto(+) **(C)** in bladder IMT.
