# Supplementary material for: A rare huge bladder inflammatory myofibroblastic tumor treated by en bloc resection with diode laser: a case report and literature review
Source: Front Oncol. 2024 Mar 11;14:1327899. doi: 10.3389/fonc.2024.1327899 (PMC10961466; doi:10.3389/fonc.2024.1327899)
Supplement: Supplementary Figure 1 — The first postoperative pathology results. (A–C) The results in IHC staining of CK(AE1/AE3) (+) (A), Desmin(+) (B), and SMA-auto(+) (C) in bladder IMT. [file Image_1.pdf]

Supplementary Material

1 Supplementary Figure

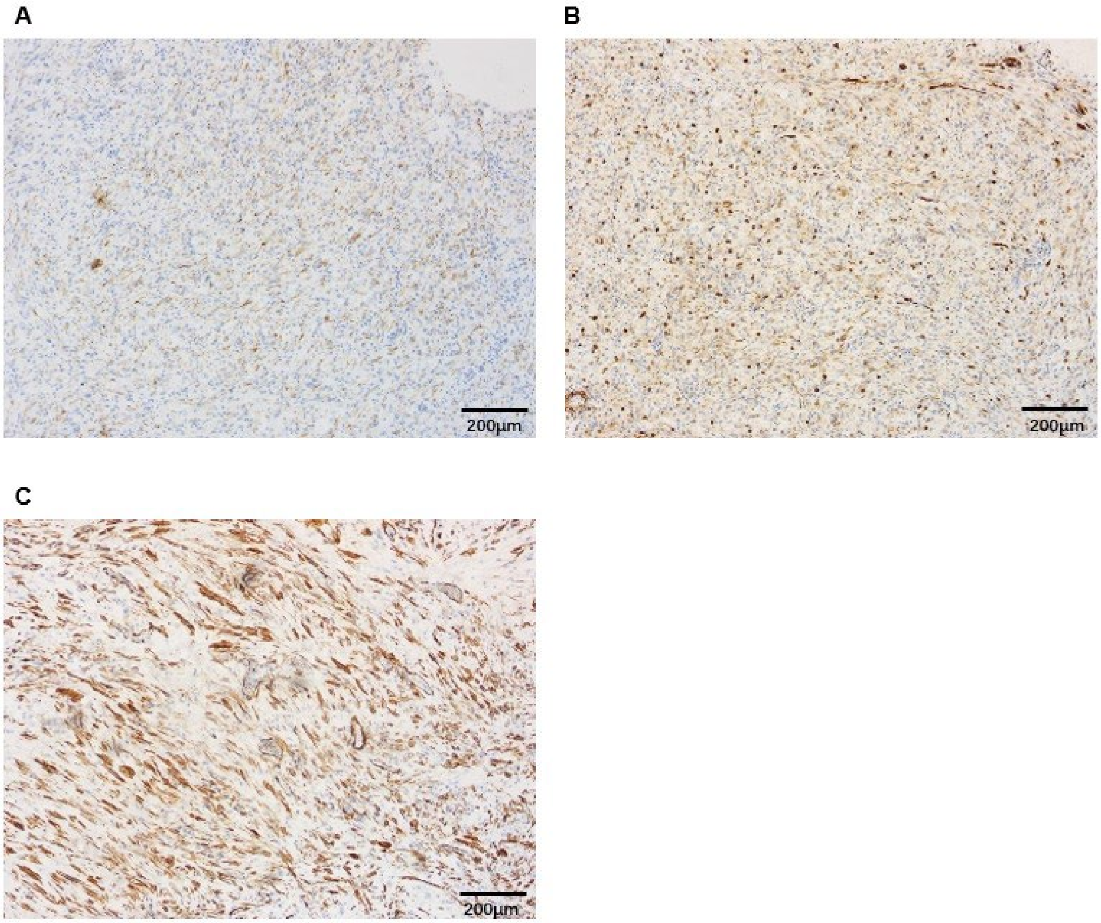

Supplement figure 1 The first postoperative pathology results. (A-C) The results in IHC staining of CK(AE1/AE3) (+) (A), Desmin(+) (B), and SMA-auto(+) (C) in bladder IMT.
