# Supplementary material for: A rare huge bladder inflammatory myofibroblastic tumor treated by en bloc resection with diode laser: a case report and literature review
Source: Front Oncol. 2024 Mar 11;14:1327899. doi: 10.3389/fonc.2024.1327899 (PMC10961466; doi:10.3389/fonc.2024.1327899)
Supplement: Supplementary file 2 [file DataSheet_1.pdf]

CARE Checklist of information to include when writing a case report

CARE case report guidelines

(cc) BY-NC-ND

Topic	Item	Checklist item description	Reported on Line
**Title**	1	The diagnosis or intervention of primary focus followed by the words "case report"	Title
**Key Words**	2	2 to 5 key words that identify diagnoses or interventions in this case report, including "case report"	Keywords
**Abstract (no references)**	3a	Introduction: What is unique about this case and what does it add to the scientific literature?	The third paragraph of the abstract
	3b	Main symptoms and/or important clinical findings	The second paragraph of the abstract
	3c	The main diagnoses, therapeutic interventions, and outcomes	The second paragraph of the abstract
	3d	Conclusion—What is the main "take-away" lesson(s) from this case?	The third paragraph of the abstract
**Introduction**	4	One or two paragraphs summarizing why this case is unique (**may include references**)	The third paragraph of the introduction
**Patient Information**	5a	De-identified patient specific information.	The first paragraph of the case description
	5b	Primary concerns and symptoms of the patient.	The first paragraph of the case description
	5c	Medical, family, and psycho-social history including relevant genetic information	The first paragraph of the case description
	5d	Relevant past interventions with outcomes	The second paragraph of the introduction
**Clinical Findings**	6	Describe significant physical examination (PE) and important clinical findings.	The first paragraph of the case description
**Timeline**	7	Historical and current information from this episode of care organized as a timeline	The fourth paragraph of the case description
**Diagnostic Assessment**	8a	Diagnostic testing (such as PE, laboratory testing, imaging, surveys).	The first paragraph of the case description
	8b	Diagnostic challenges (such as access to testing, financial, or cultural)	The fourth paragraph of the discussion
	8c	Diagnosis (including other diagnoses considered)	The second paragraph of the case description
	8d	Prognosis (such as staging in oncology) where applicable	The fourth paragraph of the case description
**Therapeutic Intervention**	9a	Types of therapeutic intervention (such as pharmacologic, surgical, preventive, self-care)	The second and third paragraph of the case description
	9b	Administration of therapeutic intervention (such as dosage, strength, duration)	The second and third paragraph of the case description
	9c	Changes in therapeutic intervention (with rationale)	The second and third paragraph of the case discussion
**Follow-up and Outcomes**	10a	Clinician and patient-assessed outcomes (if available)	The fourth paragraph of the case description
	10b	Important follow-up diagnostic and other test results	The fourth paragraph of the case description
	10c	Intervention adherence and tolerability (How was this assessed?)	The second paragraph of the discussion
	10d	Adverse and unanticipated events	The last paragraph of the discussion
**Discussion**	11a	A scientific discussion of the strengths AND limitations associated with this case report	Discussion
	11b	Discussion of the relevant medical literature **with references**.	The first to seventh paragraphs discussion
	11c	The scientific rationale for any conclusions (including assessment of possible causes)	Discussion
	11d	The primary "take-away" lessons of this case report (without references) in a one paragraph conclusion	Conclusion
**Patient Perspective**	12	The patient should share their perspective in one to two paragraphs on the treatment(s) they received	The fourth paragraph of the case description
**Informed Consent**	13	Did the patient give informed consent? Please provide if requested	Yes ☑ No ☐
